# CsAGP: Detecting Alzheimer’s disease from multimodal images via dual-transformer with cross-attention and graph pooling

**DOI:** 10.1016/j.jksuci.2023.101618

**Published:** 2023-06-24

**Authors:** Chaosheng Tang, Mingyang Wei, Junding Sun, Shuihua Wang, Yudong Zhang, Alzheimer’s Disease Neuroimaging Initiative

**Affiliations:** a School of Computer Science and Technology, Henan Polytechnic University, Jiaozuo, Henan 454000, PR China; b School of Computing and Mathematical Sciences, University of Leicester, Leicester LE1 7RH, UK; c Department of Information Systems, Faculty of Computing and Information Technology, King Abdulaziz University, Jeddah 21589, Saudi Arabia

**Keywords:** Alzheimer’s disease, Vision transformer, Multimodal image fusion, Deep learning

## Abstract

Alzheimer’s disease (AD) is a terrible and degenerative disease commonly occurring in the elderly. Early detection can prevent patients from further damage, which is crucial in treating AD. Over the past few decades, it has been demonstrated that neuroimaging can be a critical diagnostic tool for AD, and the feature fusion of different neuroimaging modalities can enhance diagnostic performance. Most previous studies in multimodal feature fusion have only concatenated the high-level features extracted by neural networks from various neuroimaging images simply. However, a major problem of these studies is over-looking the low-level feature interactions between modalities in the feature extraction stage, resulting in suboptimal performance in AD diagnosis. In this paper, we develop a dual-branch vision transformer with cross-attention and graph pooling, namely CsAGP, which enables multi-level feature interactions between the inputs to learn a shared feature representation. Specifically, we first construct a brand-new cross-attention fusion module (CAFM), which processes MRI and PET images by two independent branches of differing computational complexity. These features are fused merely by the cross-attention mechanism to enhance each other. After that, a concise graph pooling algorithm-based Reshape-Pooling-Reshape (RPR) framework is developed for token selection to reduce token redundancy in the proposed model. Extensive experiments on the Alzheimer’s Disease Neuroimaging Initiative (ADNI) database demonstrated that the suggested method obtains 99.04%, 97.43%, 98.57%, and 98.72% accuracy for the classification of AD vs. CN, AD vs. MCI, CN vs. MCI, and AD vs. CN vs. MCI, respectively.

## 1 Introduction

Alzheimer’s disease (AD) and its prodromal stage, mild cognitive impairment (MCI), are the primary causes of dementia. The increasing impairment of memory and cognitive abilities differentiates AD and MCI. Between 2000 and 2019, the number of people who passed from AD increased by more than 145% in the United States in 2019 (Alzheimer’s disease facts and figures, 2022). More than 11 million Americans are offering unpaid caregiving of around 16 billion hours worth $271.6 billion to people with AD in 2021 (Alzheimer’s disease facts and figures, 2022). The report shows that the global burden of AD will reach $2 trillion, and 152 million people will suffer from AD by 2050 (Patterson, 2018). There is no effective drug or method of curing AD for this complicated pathogenesis (Liu, 2020). Consequently, precise early detection and treatment of AD are of utmost importance.

Generally, according to different pathological features, the disease has three stages: control normal (CN), MCI, and AD. Neuropsychological tests and neuroimaging diagnoses are the primary clinical examination methods for AD. The mini-mental state examination (MMSE) and the clinical dementia rating (CDR) are the most commonly utilized tools for clinical neuropsychological evaluation of AD and assist doctors in determining the stage of a patient. With medical technology’s rapid advancement, neuroimaging has become the mainstream method for diagnosing AD. Due to the great precision presentation of brain tissue and the capacity to differentiate between grey and white matter, magnetic resonance imaging (MRI) has turned into the common tool for neuroimaging diagnosis of AD. positron emission tomography (PET), another widely adopted neuFroimaging tool for diagnosing AD, may detect the spread of lesions and alterations in glucose metabolism using imaging agents. Moreover, the fusion of complementary information provided by different neuroimaging modalities further improves AD’s diagnostic performance.

In the past decades, inspired by deep learning in the field of computer vision, deep learning methods have been extensively employed in AD Computer-Aided Diagnosis (CAD) (Suk et al., 2014; Liu et al., 2023). However, most methods only utilized unimodal images as input, and information provided by unimodal images is one-sided, which may lead to suboptimal performance for AD diagnosis. Researchers have recently shown increasing interest in multimodal images for AD diagnosis, and more deep learning-based multimodal feature fusion algorithms have been created (Kong et al., 2022; Zhang et al., 2019). Specifically, according to the type of input modalities, these algorithms can be split into four classes: the raw image-based methods, the fused image-based methods, the generated image-based methods, the neuroimaging, and clinical data-based methods. The raw image-based methods feed the multi-input neural networks with the raw neuroimaging images or their preprocessed images, then fuse different modal features by latent representation learning (Zhang and Shi, 2020; Meng, 2022). Although these methods are simple to implement, they are prone to causing excessive model parameters and ignoring the interaction of information between modalities. The fused image-based methods merge important and discriminative information from several modalities to a sole fused image through image preprocessing steps to reduce model parameters, then take the sole fused image as model input (Song et al., 2021; Wu, 2018). However, these preprocessing steps are time-consuming and also increase computational costs. Due to factors such as cost or availability, multimodal images are not always fully realized in practice. To address this limitation and utilize incomplete data, the generated image-based methods directly generate missing data from an available modality through image generation algorithms such as generative adversarial networks (GANs) (Pan and Wang, 2206; Logan, 2021). Regrettably, it is difficult to analyze the generated images quantitatively due to the particularity of medical images.

On the other hand, neuroimaging and clinical data-based methods combine neuroimaging and clinical data to simulate the diagnostic process of clinicians (Zhao et al., 2019; Lin et al., 2021). Even though this method can increase the performance of AD diagnosis even further, it suffers from the same limitation of time-consuming preprocessing steps for clinical data. Furthermore, extracting effective features from high-dimensional gene sequences is challenging.

Although convolutional neural networks (CNNs) ’s convolutional operation improves their ability to capture local information, this generally results in CNNs learning features that are only relevant to nearby brain regions rather than more generalizable features that can be applied across multiple brain regions. It has been found that even distant brain regions can have significant interactions. Hence AD-related disorders can affect many different brain parts (Lyu et al., 2022). A new architecture based on the self-attention mechanism, vision transformer (ViT), was designed to effectively model global context without layering hierarchical convolution layers. ViT is powerful in classifying AD in several investigations (Zhu, 2022; Kushol et al., 2022). Notably, the problem of token redundancy (Rao et al., 2021) in ViT without taken into account in their models.

Additionally, from the point of view of multimodal feature fusion strategy, most existing multimodal data fusion diagnosis methods purely combine high-level selected features from the various modalities to merge their information, ignoring the fusion of low-level features. Compared to high-level features, low-level features have higher resolution and contain more location and detail information which is equally important for AD diagnosis. On the other hand, feature extraction and fusion stages are performed independently in these methods, ignoring the cross-modal interactions, which restricts the model from learning a shared representation (Khan et al., Jun. 2021). Cross-modal interaction has been shown to fully fuse features and further improve model performance (Tan and Bansal, 2019).

In this paper, we design a dual-transformer based on cross-attention and graph pooling algorithm (CsAGP) to solve the above issues, which enables multi-level feature interaction between the input modalities through the cross-attention mechanism. Specifically, we first construct a dual-branch framework for extracting multimodal features and disease classification. Then, to learn rich fused features, an innovative cross-attention fusion module (CAFM) is built to extract and fuse multimodal features based on the self-attention mechanism. To reduce token redundancy in the proposed model, a concise Reshape-Pooling-Reshape (RPR) frame-work was developed to select tokens of high significance via a graph pooling algorithm while avoiding high computation and memory costs. The proposed CsAGP has performed satisfactorily in the Alzheimer’s Disease Neuroimaging Initiative (ADNI) database. Our major contributions are as follows:

(1) A dual-branch vision transformer with cross-attention and graph pooling algorithm, called CsAGP, is present to model the global information of images based on the pure self-attention mechanism to detect multimodal fused features for AD diagnosis.
(2) An innovative cross-attention mechanism-based multi-modal feature fusion method is suggested, which can efficiently learn a shared feature representation of MRI and PET images.
(3) A concise Reshape-Pooling-Reshape (RPR) framework is developed, which filters tokens based on a graph pooling algorithm to reduce computation costs and token redundancy in the proposed model.

## 2 Related work

This section first introduces the current deep learning-based multimodal AD diagnosis methods. Generally, based on the type of input modalities, these methods can be split into four classes: (i) the raw image-based methods, (iii) the fused image-based methods, (iii) the generated image-based methods, and (iv) the neuroimaging clinical data-based methods. Then, an introduction to vision transformers for AD diagnosis is described.

### 2.1 Deep learning-based multimodal AD diagnosis

The raw image-based methods input raw neuroimaging images of different modalities or their preprocessed images into multi-input neural networks to fuse features between modalities by latent representation learning. Fang et al. (Fang et al., 2020) employed three CNNs (GooleNet, ResNet, and DenseNet) with a dropout mechanism and the Adaboost ensemble algorithm to improve AD’s classification precision. They built a stack of CNNs to learn multimodal representations from MRI and PET images while utilizing the Adaboost ensemble algorithm to fuse their probabilistic scores. In their model, the dropout mechanism is utilized to exclude the slices with poor discrimination. However, the Adaboost ensemble algorithm prioritized misclassification data, which could lead to a bias due to noise data.

Adaptive-similarity-based multimodal feature selection (ASMFS) was developed by Shi et al. (Shi, 2022); which combines adaptive similarity learning with feature selection. Unfortunately, they only checked the efficacy of their model for binary classification problems and did not test it for multi-class situations. Jiao et al. (Jiao et al., 2022) devised a multimodal feature selection approach (FC2FS), which generates feature equivalence regularization and feature construction regularization through the similarity matrix calculated from the multimodal feature vertices. Finally, a support vector machine (SVM) is employed to finish the process of AD diagnosis. It is possible that the model’s generalization ability was not maximized because only standard techniques of generating correlation coefficients were used throughout the construction of the similarity matrix. Zhang et al. (Zhang et al., 2021) developed a 2.5D CNN-based framework that extracts 2.5D patches from the hippocampal areas of MRI and PET images. Then, these 2.5D patches are integrated by a training approach termed branching pre-training to provide a full AD diagnosis.

Although the above methods can further raise the accuracy of AD diagnosis compared with traditional machine learning methods (Shi et al., 2019; Richhariya et al., May 2020), multi-input neural networks demand a lot of model parameters and computational costs. In addition, since only the high-level features of different modalities are concerned, the latent representation learning over-looks feature interactions between modalities. The fused image-based methods integrate important and discriminative information from several modalities into a sole fused image based on image fusion algorithms and then take the fused image as the model’s input to address these limitations. Song et al. (Song et al., 2021) acquired a new neuroimaging modality famous as “GM-PET” by fusing gray matter (GM) of 3D structural MRI and PET images. Experimentally, their method can improve accuracy by up to 16.48% compared to the unimodal. Although their method significantly reduces the model’s parameters compared to other multi-modal fusion methods, the preprocessing steps are time-consuming.

On the other hand, Kang et al. (Kang et al., 2020) obtained fractional anisotropy (FA) and mean diffusivity (MD) 2D image slices from diffusion tensor imaging by FMRIB Software Library (FSL), then merged them with the corresponding index MRI image slices into an RGB image, finally fed the RGB image into the VGG network to complete the classification of MCI and CN. However, they only tested their method on the CN vs. MCI task and did not consider diagnostic tasks involving other stages, such as AD. To avoid the problem that 2D slices will lose image-spatial information of raw 3D images, similar to Ref. (Song et al., 2021). Kong et al. (Kong et al., 2022) fused the GM into a 3D GM image and then fed the 3D GM image into a 3D CNN. Finally, they got 93.21% accuracy on AD vs. CN. Although the above methods can reduce the amount of computation compared to multi-input neural networks, the pre-processing steps of image fusion are demanding.

In practice, multimodal images may be incomplete for high financial costs or availability. To address this limitation and utilize incomplete data, with generative adversarial networks (GANs), the generated image-based methods directly produce missing data from a present modality. By combining a GAN and a dense CNN, Gao et al. (Gao et al., 2022) constructed a hybrid framework (PT-DCN) to diagnose AD. To make use of multimodal data, they generate PET images by the task-induce pyramid GAN. The PT-DCN can learn and merge multimodal features gradually. However, their experiment data was derived from ADNI-1 and ADNI-2, which may affect the experimental accuracy by varying MR scanner parameters. Zhang et al. (Zhang et al., 2022) developed a 3D GAN (BPGAN) to generate 3D PET images from MRI images. They devised a cutting-edge hybrid loss function to keep tabs on the brain data training process. In the end, they obtained an accuracy of 98.11% for AD vs. CN. Ye et al. (Ye et al., 2022) developed a paired GAN, which uses deep MRI features extracted by a feature extractor. The network can produce equivalent PET features in place of raw MRI images to reduce the model’s size.

While the previous work has proven that generating missing data for AD diagnosis is possible, it has certain drawbacks when synthesizing multimodal medical images. First, the trustworthiness of the generated data is a serious issue. There are obvious differences between synthetic and real images regarding semantics and resolution because of the complicated spatial structure of medical images. Second, erratic training methods. The visual pattern in medical images is often unclear. Since GAN’s training processes are prone to instability (Creswell et al., 2018), it is difficult to spot erratic behavior and implausible outcomes. At last, the evaluation is not always convincing. Because of the disclosure of ground-truth images, typical pixel-wise metrics have trouble quantitatively evaluating generated images.

The clinical diagnosis of AD relies on neuroimaging data but also the subject’s clinical and biochemical information. It can significantly increase the accuracy of AD diagnosis by fusing with clinical and neuroimaging data. Zhang et al. (Zhang et al., 2019) employed two separate CNNs to analyze MRI and PET images for diagnosing AD. They suggested a method based on the Pearson coefficient that combines the neuroimaging diagnostic with neuropsychological evaluations (MMSE and CDR) to steer the output of their model. However, they focused solely on the high-level features of various modal images and paid little attention to the interactions of the low-level features.

Tu et al. (Tu et al., 2022) created an innovative multimodal AD diagnostic model. They first suggested a geometric; algebraic approach that extended low-dimensional clinical data of subjects, such as profiles, gene sequences, and MMSE scores, to high-dimensional features at various levels. Second, according to the degree of influence, the feature filtration algorithm eliminates irrelevant features from high-dimensional features and yields transformed ones. Finally, the transformed features are combined with those extracted by CNNs from MRI images. Nan et al. (Nan, 2022) suggested a framework to investigate the impact of different modalities and their combinations on AD diagnosis. Ultimately, they found that with the addition of different modal data, the diagnostic performance of AD increased gradually. Furthermore, they discovered that adding single nucleotide polymorphism (SNP) data could bring a 3% to 7% performance boost to the AD diagnostic.

### 2.2 Vision Transformer-Based AD diagnosis

Rather than stacking hierarchical convolution layers, the vision transformer successfully models the image’s global context based on the self-attention mechanism. Several works have shown the potential of vision transformers in AD diagnosis. Lyu et al. (Lyu et al., 2022) transferred a pre-trained ViT to the brain imaging dataset. They employed ViT as the backbone network and 2D MRI images as input and finally got 95.3% accuracy in AD diagnosis. Zhu et al. (Zhu, 2022) merged representation learning, feature distillation, and classification into a coherent model termed Brain Informer (BraInf). They initially deployed a multi-head ProbSparse self-attention block to minimize computational costs for representation learning. Later, a structural distillation block was utilized to underrate the dimension of the three-spatial tensor, which further reduces computational costs. However, the patch size of MRI images was predetermined in their experiments, which is ill-considered as the structural changes within every region produced by AD are not fixed.

On the other hand, Jang et al. (Jang and Hwang, 2022) developed a medical classifier for diagnosing AD. They trained a 3D CNN to recover local features linked to anomalies of AD from 3D MRI images and then fed the obtained local features into a transformer block to combine multi-plane and multi-slice features. This procedure can mark a general representation in 3D MRI images. They achieved 93.21%, 93.27%, and 85.26% accuracies on the ADNI, AIBL, and OASIS datasets. Xing et al. (Xing et al., 2022) assembled a block to transpose the 3D PET images into 2D images and fed the transposed image into a paralleled vision transformer model for AD diagnosis.

In general, deep learning-based multimodal AD diagnosis methods can automatically extract the AD-related features from complex neuroimaging images via CNNs without domain-specific knowledge, which can avoid errors caused by artificial. However, it is difficult to capture global features that across brain regions for CNNs. Meanwhile, although the vision transformer-based methods can model image-global information by the self-attention mechanism, most works do not consider the problem of token redundancy in their models. In this paper, we proposed a dual-transformer that fuses MRI and PET image features based on the cross-attention mechanism and selects discriminative tokens using a graph pooling algorithm to reduce redundancy.

## 3 Materials

Both the database ADNI and the image preprocessing pipelines are detailed in this section.

### 3.1 Datasets

Data used in this article were obtained from ADNI, which was settled in 2003 as a public–private alliance. The ADNI aims to develop clinical, imaging, and genetic to diagnose AD. Following the methodology described in Ref. (Golovanevsky et al., 2206), 766 subjects from the ADNI1/GO and ADNI2 phases were selected, including MRI and PET images. The numbers of AD, MCI, and CN subjects were 214, 226, and 326, respectively. There includes a T1-weighted MRI and a PET (FDG-PET) image in a NIfTI file format for every subject. Table 1 shows the clinical information (e.g., sex, age, MMSE scores, and CDR scores) of selected subjects. MRI images of subjects in this paper were acquired by three MR scanners, SIEMENS, Philips Medical Systems, and GE Medical Systems.

The imaging parameters are, respectively, a) repetition time [TR]= 3000ms, echo time [TE]= 3.5ms, inversion time [TI]= 1000ms, flip angle = 8°, thickness = 1.2mm, matrix size = 192 × 192 × 160, field strength = 3.0T. b) [TR] = 6.8005ms, [TE]= 3.116ms, [TI] = 0ms, flip angle = 9°, thickness = 1.2mm, matrix size = 256 × 256 × 170, field strength = 3.0T. c) [TR]= 7.332ms, [TE]= 3.036ms, [TI]= 400ms, flip angle = 11°, thickness = 1.2mm, matrix size = 256 × 256 × 196, field strength = 3.0T. The ADNI data acquisition details can be seen on the official webpage of ADNI.^2^

### 3.2 Data preprocessing

To remove the impact of various imaging parameters, the raw images were preprocessed using a normal preprocessing method described in Ref. (Suk et al., 2014) by the FMRIB Software Library (FSL)^3^ and Advanced Normalization Tools (ANTs).^4^

First, the acpcdetect software^5^ shifted all of the raw MRI images to the exact center of the anterior commissure (AC) to the posterior commissure (PC) dividing line. After adjustment of force inhomogeneity by the nonparametric non-uniform force normalization (N4) algorithm, these MRI images were processed through the Brain Extraction Tool (BET) in the FSL to delete the cerebellum and skull. Second, we ensured that the skulls were clean and the dura was gone by hand-checking the images. Finally, all the preprocessed MRI images were spatially normalized onto a standard space.

**Fig. 1:**
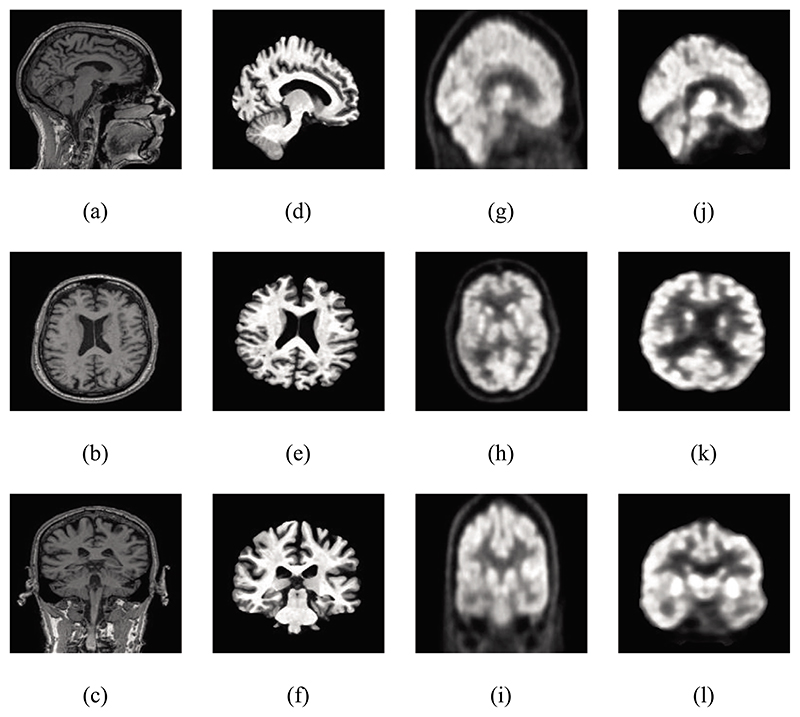
Contrasting of the raw and preprocessed images.

PET images were precisely aligned with their corresponding MRI images. The Gaussian kernel was used to further smooth the preprocessed images. Utilizing the med2image tool,^6^ 181 MRI and PET axial view slice images were acquired, respectively. Only slices with indices 80–100 have been used in this paper, as these images contained the most relevant information for the whole brain. To meet the input specifications, these slice images were scaled to 224×224. The images before and after preprocessed are shown in Fig. 1.

## 4 Methods

**Fig. 2:**
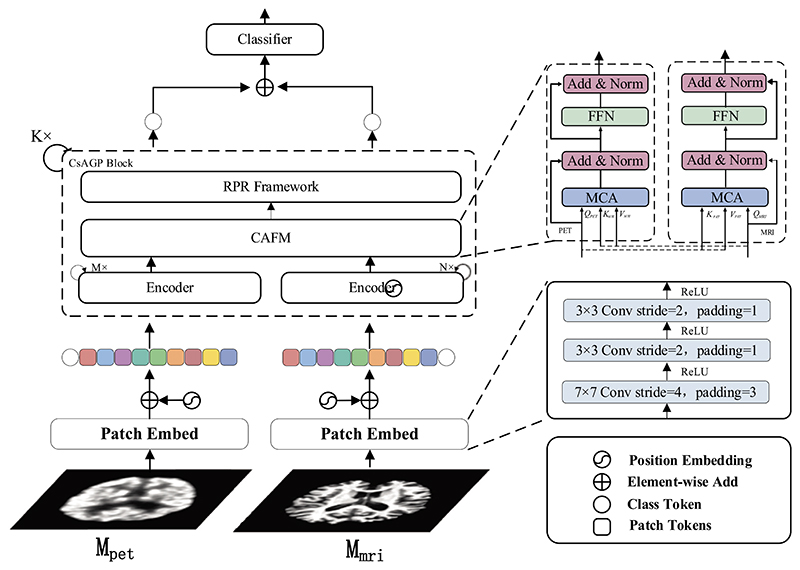
An illustration of the proposed CsAGP.

Considering the difference in resolution and information in MRI and PET images, we designed two branches of different computational complexity by the encoder block proposed in Ref. (Dosovitskiy, et al., 2010) to process MRI and PET images individually. The proposed CsAGP, shown in Fig. 2, composes of three components: (i) two identical Patch Embed modules are implemented to convert MRI and PET images into non-overlapping patch tokens, respectively, (ii) A stack of K CsAGP Blocks that output the final feature representation for each modality, (iii) a classifier that predicts AD stage based on the shared feature representation.

The main implementation steps of our model can be described as follows. Firstly, the Patch Embed module is carried out on 2D MRI and PET images, which splits and transposes the input image into a series of patch tokens with a fixed size. Then the positional encoding and the class token are added to each token sequence. Then, these token sequences with positional encoding are passed into the CsAGP Block as image feature sequences. The feature sequences first pass through the Encoder module, which primarily consists of the self-attention mechanism and a feed-forward network (FFN). Compared to CNNs, the self-attention mechanism can efficiently model long-range relationships (Dosovitskiy, et al., 2010). Secondly, the outputs of the Encoder module are fed into the CAFM for multimodal feature fusion. The CAFM realizes the interactions of multi-level features through a pure self-attention mechanism which is different from the previous methods (Zhang et al., 2019) that concatenates the high-level features into a long vector. After that, the fused token sequences are passed through the RPR framework, which selects the discriminative tokens through a graph pooling algorithm to reduce token redundancy and memory costs. Finally, the class tokens of each modality sequence as an agent are combined to get the shared feature representation as the output of CsAGP, as detailed in the following subsections.

### 4.1 Patch Embed

In ViT, the original image is directly converted into fixed-size patches by linear projections alone, which is a poor way to capture low-level information in images. To overcome this limitation, as shown in Fig. 2. A novel tokenization approach was employed to make optimal use of CNN’s strength in retrieving low-level features and minimizes the training difficulty of embedding by decreasing the patch size. Specifically, for M_mri_ branch, given an input image **x***_mri_* ∈ ℝ^3×*H×W*^, to minimize the size of input images, we first utilize a 7 × 7 convolution with a stride of 4 and a padding of 3, then two additional 3 × 3 convolutions with a stride of 2 and padding of 1, for improved low-level information extraction.

After that, the output ${{\text{x}}^{\prime }}_{mri}\in {\mathbb{R}}^{D\times \frac{H}{P}\times \frac{W}{P}}$ of the Patch Embed module is flattened and transposed to get the patch tokens matrix $x_{patch\;}^{mri}\in {\mathbb{R}}^{N\times D},$ where *N = HW/P*^2^ is the number of patches, D is the number of enriched channels, (H, W) and (P, P) represent the resolution of the input images and image patches, respectively. Finally, the positional encoding and an extra class token $x_{cls}^{mri}\in {\mathbb{R}}^{1\times D}$ are added as image representations to the patch tokens matrix ${\text{x}}_{\text{patch}\;}^{\text{mri}}\text{,}$ resulting in the final patch tokens matrix $x_{f}^{mri}\in {\mathbb{R}}^{(N+1)\times D}$ for further steps. These procedures can be noted as follows:

(1) $${{\text{x}}^{\prime }}^{mri}=\text{ReLU}(\text{Conv3}(\text{ReLU}(\text{Conv2}(\text{ReLU}(\text{Conv1}(x_{mri}))))))$$

(2) $$x_{patch\;}^{mri}=\text{Transpose}(\text{Flatten}({{\text{x}}^{\prime }}^{mri}))$$

(3) $$x_{f}^{mri}=[x_{cls}^{mri}∥x_{patch\;}^{mri}]+\text{PE},\;\text{PE}\in {\mathbb{R}}^{(N+1)\times D}$$

where ∥ is the concatenate operation and *PE* ∈ ℝ^(*N*+1)×*D*^ represents the positional encoding following Ref. (Dosovitskiy, et al., 2010). The *M_pet_* branch follows the same procedures but takes a 2D PET image as input and adds another class token ${\text{x}}_{cls}^{pet}\in {\mathbb{R}}^{1\times D}.$

### 4.2 Cross-Attention fusion module (CAFM)

The cross-attention fusion module (CAFM) was designed to fuse multimodal features efficiently. Specifically, let $x_{f}^{i}\in {\mathbb{R}}^{(N+1)\times D}$ be the final patch tokens matrix output from the previous step at branch i, where i represents the i-th branch (*M_mri_* or *M_pet_*).

Fusion in the CAFM involves the class token ${\text{x}}_{\text{cls}}^{\text{i}}$ from one branch and the patch tokens ${\text{x}}_{\text{patch}\;}^{\text{i}}$ from another branch. Specifically, the class token ${\text{x}}_{\text{cls}}^{\text{i}}$ is utilized as an agent to share information between the patch tokens ${\text{x}}_{patch\;}^{i}$ from another branch, and then the class token ${\text{x}}_{\text{cls}}^{\text{i}}$ returns to the i-th branch so that it combines the multimodal features efficiently and favorably. Following the fusing of patch tokens from another branch, the class token exchange information with its own patch tokens once more in the subsequent blocks to impart the information obtained from another branch into its own patch token representations.

**Fig. 3:**
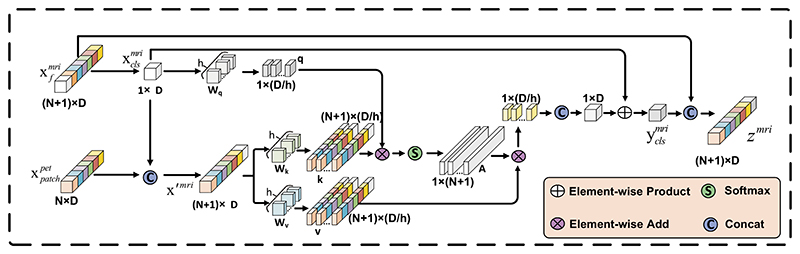
Multi-heads cross-attention feature fusion for M_mri_ branch.

As shown in Fig. 2. The final matrix ${\text{x}}_{\text{f}}^{\text{i}}$ is entered into the CAFM, which includes two sub-blocks. Each sub-block has two parts. The first part main contains a multi-heads cross-attention (MCA) mechanism to swap information between the patch tokens ${\text{x}}_{\text{patch}}^{\text{i}}$ from another branch. An exemplification of the MCA on the M_mri_ branch is proved in Fig. 3. For M_mri_ branch, it first collects the patch tokens $x_{patch\;}^{pet\;}\in {\mathbb{R}}^{N\times D}$ from the M_pet_ branch, and then concatenates them with own class token ${\text{x}}_{\text{cls}}^{\text{mri}}\text{,}\;$ as expressed in Eq. (4):

(4) $${{\text{x}}^{\prime }}^{\;mri}=\left[{\text{x}}_{cls}^{mri}\|{\text{x}}_{patch}^{pet}\right]$$

Then, the module performs the MCA between ${\text{x}}_{\text{cls}}^{\text{mri}}$ and ***x***′ ^*mri*^, where class token ${\text{x}}_{\text{cls}}^{\text{mri}}$ of M_mri_ branch is the query as patch-token information has already been integrated into the class token. The MCA could be written mathematically as:

(5) $$q=x_{cls}^{mri}W_{q},k={x^{\prime }}^{\;mri}W_{k},v={x^{\prime }}^{\;mri}W_{v}$$

(6) $$A=\text{softmax}(qk^{T}/\sqrt{D/h})$$

(7) $$\text{MCA}({{\text{x}}^{\prime }}^{\;mri})=\text{Av}$$

where **W**_*q*_, **W**_*k*_, **W**_*v*_ ∈ ℝ^*D*×(*D*/*h*)^ are learnable parameters, D is the embedding dimension of tokens, h represents the number of heads. Because only the class token is utilized in the queries, the computational and memory costs of MCA are linear instead of quadratic in constructing A. Finally, the output z^mri^ of the first part with a residual shortcut is defined as follows:

(8) $$y_{cls}^{mri}=x_{cls}^{mri}+\text{MCA}([(x_{cls}^{mri}∥x_{patch}^{pet}])$$

(9) $$z^{mri}=[y_{cls}^{mri}∥x_{patch\;}^{mri}]$$

The second part primarily consists of a feed-forward network with non-linear activation, which performs a spatial transformation of z^mri^ by two linear projecting layers to enhance the representation ability of tokens. It can be described as follows:

(10) $${\text{Z}}^{mri}=\text{LN}(\text{FFN}(\text{LN}(z^{mri}))+z^{mri})$$

(11) $$\text{FFN}(x)=\sigma (x{\text{W}}_{1}+{\text{b}}_{1}){\text{W}}_{2}+{\text{b}}_{2}$$

where W_1_ ∈ ℝ^D×K^ is the weight of the first layer, projecting each token in a higher dimension K. And W_2_ ∈ ℝ^K×D^ is the weight of the second layer. b_1_ ∈ ℝ^1×K^ and b_2_ ∈ ℝ^1×D^ are the biases. LN represents the layer normalization, σ(·) is a non-linear activation function.

### 4.3 RPR framework

**Fig. 4:**
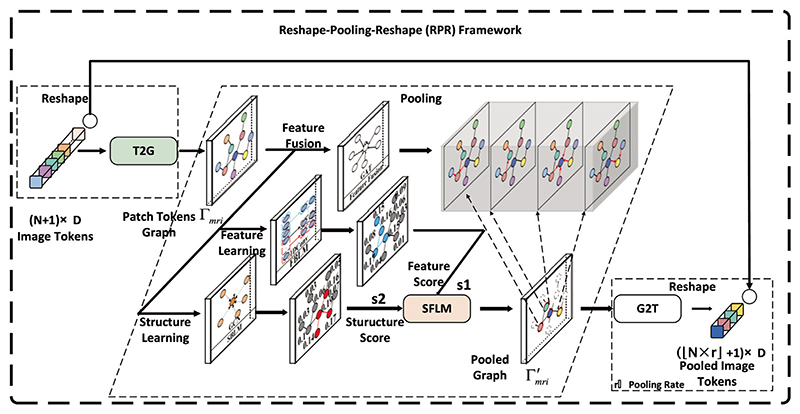
The illustration of the RPR framework of the M_mri_ branch.

To reduce token redundancy in the proposed CsAGP, we developed the Reshape-Pooling-Reshape (RPR) framework, which consists of three stages: (i) tokens to graph (T2G), (ii) graph pooling, (iii) graph to tokens (G2T), as illustrated in Fig. 4. The token sequences were converted into graph-structured data in the T2G stage. A graph pooling algorithm is utilized to filter the tokens, and only the discriminative tokens are retained. Finally, the pooled subgraph vertices are reconverted to a token sequence in the G2T stage for the next step.

#### 4.3.1 Tokens to graph (T2G)

For the M_mri_ branch, given tokens *Z^mri^* ∈ ℝ^(*N*+1)×*D*^ generated from the CAFM, we first split them into patch tokens matrix ${\text{z}}_{\text{patch}\;}^{\text{mri}}\in {\mathbb{R}}^{\text{N}\times \text{D}}$ and a class token ${\text{z}}_{\text{cls}\;}^{\text{mri}}\in {\mathbb{R}}^{1\times \text{D}}$ accordingly. Then, a graph $G_{\text{mri}}=(V,\text{A})$ is constructed, where V represents the vertex set consisting of vertices {*v_1_*, …, *v_N_*}, and A ∈ {0, 1}^N×N^ is the adjacency matrix describing the edge connection information of $G_{\text{mri}}.$

In other words, a graph $G_{\text{mri}}$ with N vertices and each vertex v_i_ in the graph has a corresponding D-dimensional feature vector ${\text{z}}_{\text{i}\;}^{\text{mri}}\in {\text{R}}^{1\times \text{D}}$ was constructed. The feature matrix ${\text{z}}_{\text{patch}\;}^{\text{mri}}\in {\mathbb{R}}^{\text{N}\times \text{D}}$ stacks N feature vectors. Then, the adjacency matrix A was established by the Euclidean distance between each vertex feature vector. Specifically, if the distance value dist_ij_ between vertices v_i_ and v_j_ is smaller than average distance μ, then A_ij_= 1, which means there is an edge between vertices v_i_ and v_j_, otherwise A_ij_= 0. The process of establishing the adjacency matrix A can be formulated as follows:

(12) $$\text{dist}=\left[\begin{array}{cccc} {\left\|{\text{z}}_{\text{1}}^{mri}-{\text{z}}_{1}^{mri}\right\|}_{2} & {\left\|{\text{z}}_{1}^{mri}-{\text{z}}_{2}^{mri}\right\|}_{2} & \cdots & {\left\|{\text{z}}_{1}^{mri}-{\text{z}}_{N}^{mri}\right\|}_{2}\\ {\left\|{\text{z}}_{2}^{mri}-{\text{z}}_{1}^{mri}\right\|}_{2} & {\left\|{\text{z}}_{2}^{mri}-{\text{z}}_{2}^{mri}\right\|}_{2} & \cdots & {\left\|{\text{z}}_{\text{2}}^{mri}-{\text{z}}_{\text{N}}^{mri}\right\|}_{2}\\ \vdots & \vdots & \ddots & \vdots \\ {\left\|{\text{z}}_{N}^{mri}-{\text{z}}_{1}^{mri}\right\|}_{2} & {\left\|{\text{z}}_{N}^{mri}-{\text{z}}_{2}^{mri}\right\|}_{2} & \cdots & {\left\|{\text{z}}_{N}^{mri}-{\text{z}}_{N}^{mri}\right\|}_{2} \end{array}\right]$$

(13) $$\mu =\frac{1}{N^{2}}\sum_{i=1}^{N}(\sum_{j=1}^{N}{\text{dist}}_{ij})$$

(14) $${\text{A}}_{ij}=\{\begin{array}{ccc} 1 & {\text{if}\;\text{dist}}_{ij}<\mu , & \\ & & 1\leq i,j\leq N\\ 0 & o\text{therwise}, & \end{array}$$

where ∥ · ∥_2_ represents the *ℓ*_2_ norm and dist indicates the distance matrix between vertices. μ is the average distances, dist_ij_ and A_ij_ are the values of distance matrix dist and A in i-th row and j-th column, respectively. Finally, the patch tokens graph $G_{\text{mri}}$ is created, where A and ${\text{z}}_{\text{patch}\;}^{\text{mri}}$ are the adjacency matrix and the feature matrix, respectively. The M_pet_ branch generates graph $G_{\text{pet}\;}$ through the same way.

#### 4.3.2 Graph pooling

we developed a novel graph pooling algorithm to reduce token redundancy by selecting the discriminative vertices of $G_{\text{mri}}$ and $G_{\text{pet}\;}$ generated in the previous stages. As shown in Fig. 4. The algorithm evaluates the importance of vertices in multiple ways. The structure-based learning module (SBLM) and the feature-based learning module (FBLM) are utilized to score vertices according to their local structure and feature information to receive scores s_1_ and s_2_, respectively. Then, the structure-feature learning module (SFLM) obtains the final score s for each vertex by combining s_1_ and s_2_. To make the final graph embedding more feature information, the vertex feature fusion module is employed to aggregate the features of the vertices to be pooled before discarding them. Finally, only the top-k vertices will be retained according to the final score s. The details of these procedures in the M_mri_ branch can be described as follows, which is the same as the M_pet_ branch.

As shown in Fig. 4, the graph $G_{\text{mri}}$ output by the T2G is fed into three branches to evaluate the importance of vertices in multiple ways. Since GCNs considers structural information of graphs, it is utilized to evaluate each vertex based on the structural information in SBLM. The mathematical representation is as follows:

(15) $$s_{1}=\sigma (W^{-\frac{1}{2}}\tilde{A}W^{-\frac{1}{2}}XW)$$

where $\tilde{A}$ and X ∈ ℝ^N×D^ are the adjacency matrix and the vertex features of the graph $G_{\text{mri}},$ respectively. W denotes the diagonal vertex degree matrix. W ∈ ℝ^D×1^ represents the learnable parameters and σ(·) is a non-linear activation function.

In FBLM, each vertex is scored by CNNs based on their feature information. It mainly consists of a 1D CNN and a Batch Normalization layer, mathematically represented as:

(16) $$s_{2}=\sigma (\text{BN}(\text{Conv}(\text{X})))$$

where X ∈ ℝ^*N*×*D*^ represents the feature matrix of the graph $G_{\text{mri}\;}.$

Then, the SFLM combines s_1_ and s_2_ to calculate the final scores of the vertices. Given the scores *s*_1_ ∈ ℝ^N×1^ and s_2_ ∈ ℝ^*N*×1^ obtained from SBLM and FBLM, respectively. First, add s_1_ and s_2_ to get a coarse score *s*′ ∈ ℝ^*N*×1^, then the coarse score *s*′ is fed into a 1D CNN to output the final scores *s* ∈ ℝ^*N*×1^. It can be denoted as:

(17) $$s=\text{BN}(\text{Conv}\;(s\prime )),\;ands\prime \;=s_{1}+s_{2}$$

After that, the vertices are sorted by the final score s, and only the top-k vertices $V\prime =\{v_{1},\cdots ,v_{k}\}$ will be retained as pooling results.

Finally, To make the final graph embedding vectors more representational, we aggregate information from neighborhood vertices in the feature fusion module with graph attention network (GAT) before discarding the vertex set $V^{\prime \prime },$ where $V^{\prime \prime }=V-V^{\prime }=\{v_{k+1},\cdots ,v_{N}\}$ represents the set of vertices that will be discarded. It can be denoted as:

(18) $$z\prime_{i}=\sigma (\frac{1}{K}\sum_{k=1}^{K}\sum_{j\in V_{i}}\alpha_{ij}^{k}W^{k}h_{j})$$

where z_i_ and h_j_ represent the feature vector and the neighbor vertices of the vertex v_i_, respectively. $V_{i}$ is the number of vertex v_i_’s adjacent vertices. *K* is the number of attention heads. $\alpha_{ij}^{k}$ is the k-th attention value between z_i_ and h_j_. W is the weight matrix.

#### 4.3.3 Graph to tokens (G2T)

Given a subgraph $G\prime_{\text{mri}}=(V\prime ,A\prime )$ of $G_{\text{mri}}$ obtained from the graph pooling stage, where $V\prime =\{v_{1},\cdots ,v_{k}\}$ and *A′* ∈ ℝ^*k*×*k*^ represent the vertex set and the adjacency matrix of $G\prime_{mri},$ respectively. Let *X′* ∈ ℝ^*k*×*D*^ denotes the feature matrix of $G\prime_{mri}.$ After the graph pooling stage, the feature matrix X′ is reassembled into token sequence ***z**′_p_* ∈ ℝ^*k*×*D*^ in G2T, then the class token ${\text{z}}_{cls}^{mri}\in {\mathbb{R}}^{1\times D}$ and a new positional encoding are added to ***z**′_p_* ∈ ℝ^*k*×*D*^ for providing spatial information, that can be expressed as follows:

(19) $$z\prime_{p}=\;\text{reshape}\;(\text{X}\prime )$$

(20) $$z_{out\;}=[z_{cls}^{mri}∥z\prime_{p}]+\text{PE},\;\text{PE}\in {\mathbb{R}}^{(k+1)\times D}$$

As shown in Fig. 2, the M_pet_ branch follows the identical operation as M_mri_ branch.

## 5 Experiment and results

In this section, the experimental setup and the results of performance evaluation measures are provided. Meanwhile, the activated area of CsAGP is visualized.

### 5.1 Experimental setup

All experiments are implemented on a workstation with two Intel Xeon Gold 6330 CPUs and four Nvidia A100 GPUs with a total of 160 GB of video memory. This workstation is equipped with Ubuntu 20.04.1 LTS. We built our model on Pytorch 1.12.0 framework and trained for 300 epochs. Adam is applied as the optimizer, and more details of experiment settings are as follows: (i) batch size is set to 128; (ii) loss function adopts the CrossEntropy; (iii); the initial learning rate is set to 1 × 10^-5^ and weight decay is set to 5 × 10^-4^. In the experimental data, 60% of the data were randomly selected for training, 20% were chosen randomly for validation, and the rest 20% of subjects were used as test data.

For CsAGP, considering the difference in resolution and information contained in MRI and PET images, Following Ref. (Chen et al., 2021); we set K= 3, M= 1, N= 3. K signifies the number of CsAGP Block, M and N indicate the number of Encoder of the PET and MRI branches, respectively. Taking into account the computation costs and benefits together as a whole, the pooling rate r is set to 0.5.

### 5.2 Performance evaluation

To provide a quantitative assessment of the effectiveness of the suggested method for diagnosing AD, several evaluation metrics, including accuracy, specificity, and sensitivity, were computed as follows:

(21) $$\text{accuracy}\;=\frac{\text{TP}+\text{TN}}{\text{TP}+\text{TN}+\text{FP}+\text{FN}}$$

(22) $$\text{sensitivity}\;=\frac{\text{TP}}{\text{TP}+\text{FN}}$$

(23) $$\text{specificity}\;=\frac{\text{TN}}{\text{FP}+\text{TN}}$$

The terms “true positive,” “true negative,” “false positive,” and “false negative” are represented as “TP,” “TN,” “FP,” and “FN,” respectively. In addition to the three criteria discussed above, the area under the curve (AUC) is another factor considered when assessing performance. The area under the receiver operating characteristic curve (ROC), sometimes known as the area under the receiver operating characteristic curve (AUC), is a performance matrix employed to measure the quality of a classifier, and a large value of AUC indicates better classification performance.

### 5.3 Experiment results

In our experiments, the whole data was divided into AD vs. CN, AD vs. MCI, CN vs. MCI, and AD vs. CN vs. MCI groups to evaluate CsAGP. Each group of experiments was conducted unimodal (MRI or PET) and multimodal (MRI and PET). To make the results more convincing, we took two identical images as the model’s input when conducting unimodal experiments. Table 2 demonstrates the comparison of the classification performances of each group.

As can be seen from Table 2, the performance of our CsAGP is outperforming the unimodal method. Specifically, the developed multimodal method obtains the classification accuracies of 99.04%, 97.43%, 98.57%, and 98.72% on AD vs. CN, AD vs. MCI, CN vs. MCI, and AD vs. CN vs. MCI, and the accuracies of MRI modality are 97.87%, 95.37%, 94.94%, and 94.21%, respectively.

Compared to MRI modality, the proposed multimodal method improves the classification performance by 1.17%, 2.06%, 3.63%, and 4.51% on AD vs. CN, AD vs. MCI, CN vs. MCI, and AD vs. CN vs. MCI, respectively. For PET modality, the accuracies on AD vs. CN, AD vs. MCI, CN vs. MCI, and AD vs. CN vs. MCI are 95.92%, 94.12%, 94.69%, and 93.37%, respectively. Compared to PET modality, the proposed multimodal method improves performance rises of 3.12%, 3.31%, 3.88%, and 5.35%, respectively. The proposed multimodal method can improve classification accuracy by combining MRI and PET significantly compared with the unimodal method.

On the other hand, it can also be found that the classification accuracy of MRI modality is surpasses PET modality in each group of classification experiments. Compared with PET modality, the accuracy of MRI increases by 1.95%, 1.25%, 0.25%, and 0.84% on AD vs. CN, AD vs. MCI, CN vs. MCI, and AD vs. CN vs. MCI, respectively. It is evident that the CsAGP can capture more discriminative features on MRI images when extracting unimodal features. We consider this is due to the high resolution of MRI images compared to PET images, which allows for better differentiation between soft tissue and anatomical structures.

**Fig. 5:**
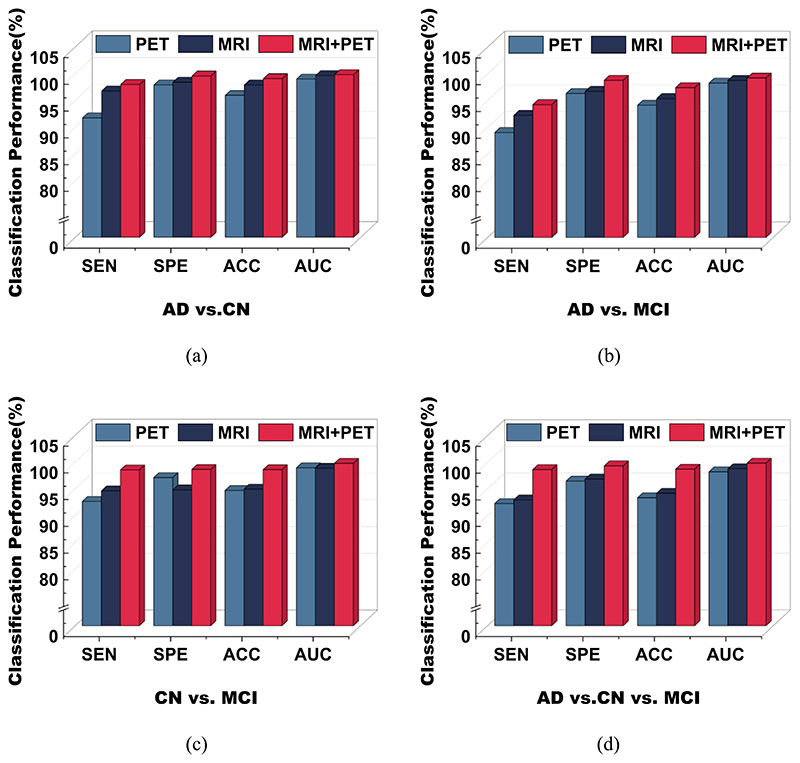
Classification performance of various groups.

Compared to the results of the other group tasks on the ADNI database, the diagnostic accuracy of the AD vs. CN task is, on the whole, higher than that of the other tasks. The same results are also in Ref. (Gao et al., 2022). This can be interpreted as AD’s primary neuroimaging features can be distinguished more easily from those of CN and MCI. Since the subtle AD-related changes that occur in MCI are not noticeable, distinguishing MCI from AD and CN only by neuroimaging data is difficult. We further present the performance of each group to demonstrate the differences between the groups intuitively. As seen in Fig. 5, the multimodal performance acts better than the unimodal, which displays that the classification performance can boost the classification efficiencies further by joining the MRI and PET modalities.

### 5.4 Comparison with other methods

In this section, we compared our CsAGP to several other multi-modal methods that are based on the ADNI database. As shown in Table 3, methods of comparison include the raw images-based methods (Zhang et al., 2019; Fang et al., 2020; Liu et al., 2022; Kun et al., 2020), the traditional machine learning method (Shi, 2022); the fused image-based method (Song et al., 2021); the generated image-based method (Zhang et al., 2022), the neuroimaging and clinical data-based method (Zhang et al., 2019).

In the AD vs. CN task, the accuracy of Fang et al. (Fang et al., 2020) was 99.27%, which is slightly larger than our suggested method. The reason is due to their utilization of ensemble learning, where the output of their model is based on three CNNs (GooleNet, ResNet, and DenseNe). By combining multiple different CNNs, they could leverage their diversity and differences. Each CNN may perform better on different subsets of data or feature subspaces. By aggregating their predictions through ensemble learning, they were able to reduce bias and variance, improving the overall accuracy of the model.

Additionally, Ref. (Fang et al., 2020) also introduced a “dropout” mechanism to discard low discrimination images, further reducing noise in their model’s input data. Although ensemble learning can enable them to achieve higher classification accuracy, training three CNNs requires many parameters and computation. In addition, compared with Fang et al. (Fang et al., 2020), CsAGP gets the best results except for accuracy.

In the AD vs. MCI task, the sensitivity metric reported by Liu et al. (Liu et al., 2022) was 94.91%, only 0.66% higher than ours, which means that the ability of their model to identify positive examples is slightly more than ours. They diagnosed AD by fusing multi-scale gray and white matter features from MRI images, while we only considered 2D slice images and single-scale feature information. By extracting features at different scales and fusing them together, the model can comprehensively utilize both local details and global contextual information, enhancing its understanding and expression capability of the images. Additionally, Ref. (Liu et al., 2022) employs the channel attention mechanism to automatically learn the importance weights of each channel, enabling the model to focus on relevant features for the task. By enhancing important channels, the model can improve its perception of crucial information, enhancing its performance.

Our CsAGP gets the best diagnostic performance in CN vs. MCI and AD vs. CN vs. MCI tasks. This can be attributed to several factors. Firstly, in addition to leveraging high-level features from different modalities, we also pay attention to the fusion of low-level features across modalities. This comprehensive integration of both high-level and low-level features enables the CsAGP to capture a more comprehensive representation of multimodal data. Secondly, by simultaneously conducting feature extraction and fusion stages for different modalities, we facilitate the effective integration of multimodal features. This simultaneous processing allows the CsAGP to learn shared representations and exploit complementary information from different modalities, further enhancing its performance. Furthermore, for reasons that the network parameters can be drastically decreased thanks to the CAFM and the RPR framework, the computational complexity and memory cost of our CsAGP does not rise.

### 5.5 Ablation experiments

Ablation experiments were carried out in this section of our CsAGP in order to demonstrate the efficacy of the CAFM and the RPR framework. To provide an accurate comparison, all experiments utilized the same settings for a fair comparison.

To reduce token redundancy and computation costs, we proposed a graph pooling algorithm to select discriminative tokens, which evaluates tokens in both feature and structural ways. Experiments were conducted to investigate the influence of the graph pooling algorithm on the prediction performance.. Since multi-classification tasks are more challenging than binary classification, the CsAGP was evaluated with different pooling rate r values. The results of the AD vs. CN vs. MCI task are reported in Table 4.

It can be seen that the classification accuracy is generally increasing with the increase of r. Specifically, when the pooling rate r increases from 0.1 to 0.5, the classification accuracy of CsAGP increases from 96.30% to 98.72%, a rise of 2.42%. However, the trend of increasing classification performance gradually flattens out when the pooling rate r is greater than 0.5. For example, when r= 0.9, the accuracy is 99.21%, only up 0.49% compared to r= 0.5. Therefore, considering computation costs and benefits together as a whole, r is set to 0.5 in our experiments.

As the pooling rate r increases, more tokens are preserved, allowing the model to capture more information and consequently leading to a rapid improvement in model performance. However, as r continues to increase, the noise and the computational cost of the model also increase. As a result, the trend of performance improvement of the model gradually flattens out.

To investigate the effectiveness of the FBLM and the SFLM, we conducted a series of experiments with different strategies. The results are listed in Table 5. Method A means using MLP to evaluate the vertex feature information (FBLM*) and linearly weighting sum vertex scores s_1_ and s_2_ (SFLM*).

By changing SFLM* to SFLM, the accuracy improves by 0.24% (Method A vs. Method B). When we change FBLM* to FBLM, the accuracy increases by 0.4% (Method A vs. Method C).Further,when using both FBLM and SFLM, as Method D, the accuracy rises by 0.49% (Method A vs. Method D). These results validate that the comprehensive consideration of both vertex position and feature information plays a crucial role in the graph pooling process. Vertex position information aids in understanding the contextual and topological relationships within the graph structure, while vertex feature information provides descriptions of vertex attributes and features, offering crucial information for vertex representation and learning. Combining these two aspects of information can assist the model in better understanding and processing graph data, enhancing the model’s performance and expressive capabilities.

To evaluate the effectiveness of CAFM in CsAGP, we removed the CAFM in the CsAGP, while other configurations remained the same. It can help us to focus on the high-level features fusion of two modalities. Comparative experiments were performed in all diagnosis tasks.

As seen from Table 6, under the influence of the CAFM, the accuracy increases by 1.33%, 1.38%, 2.9%, and 3.02% on AD vs. CN, AD vs. MCI, CN vs. MCI, and AD vs. CN vs. MCI, respectively. These results indicate that fusing multi-level features from different modalities can further improve model performance. High-level features often contain more abstract and semantically rich information, capturing the high semantics and contextual information of images.

On the other hand, low-level features focus more on low-level details and local features. By fusing multi-level features, it is possible to fully utilize the complementarity of high-level and low-level features, providing a more comprehensive and rich feature representation, and enhancing the model’s understanding and expressive capability. Furthermore, high-level features are usually less sensitive to modality differences, while low-level features are more sensitive to such differences. By integrating multi-level features, the impact of modality differences can be reduced, enhancing the model’s robustness and generalization ability towards multimodal images.

In addition, every branch of the transformer in our model develops the class token as an agent, which can exchange information between branches by the cross-attention mechanism. This makes it possible to generate attention maps in linear time rather than quadratic time.

### 5.6 Visualization

**Fig. 6:**
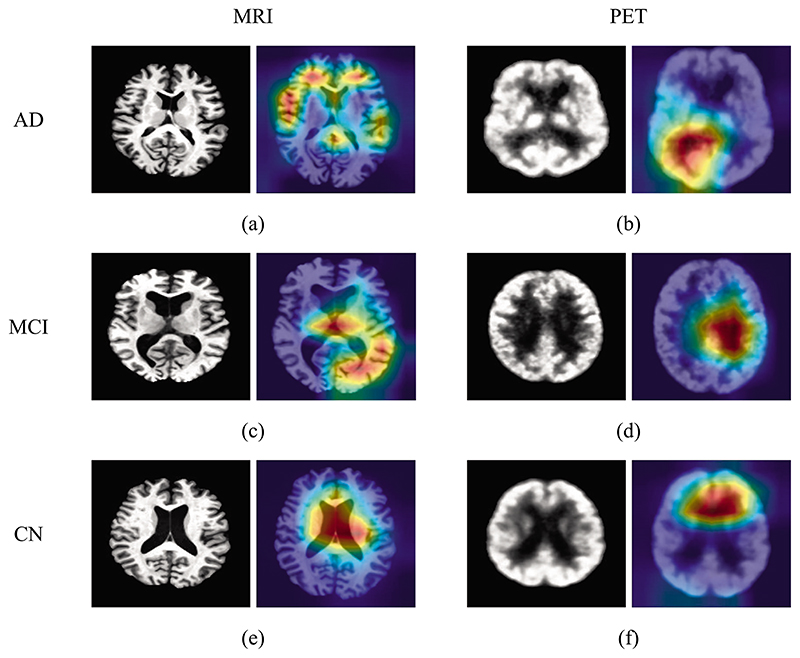
AD-related visualization map results using Grad-CAM.

Fig. 6 shows the activated areas of our CsAGP by the Grad-CAM technology (Selvaraju et al., 2017). The images on each cell’s left and right sides represent a slice image of the subject in various modalities, and the AD-related activation maps corresponded with the relevant slice image. From Fig. 6(a), it is seen from the heatmap that the areas of interest are dispersed throughout the brain. It means that our model can analyze abnormalities throughout the brain that are related to AD.

Compared with CNNs, transformer-based networks with a high receptive field have various advantages, one of which is the presence of wide activated areas. In addition, compared with AD, the heatmap areas of MCI (Fig. 6(c)) are relatively concentrated, which may be because MCI is the prodromal stage of AD with few lesion areas. The heatmap areas of CN (Fig. 6(e)) are mainly focused on the center of the brain.

Furthermore, due to different imaging protocols and information emphases, the heatmap areas of the three stages of PET images (Fig. 6(b), Fig. 6(d), and Fig. 6(f)) are relatively concentrated. It can be seen that the heatmap areas of different stages focus on different brain regions. This result further proved the view in Ref. (Suk et al., 2014) that complementary information can be obtained from a variety of modalities to improve AD diagnostic performance.

## 6 Conclusion

This paper proposes a dual-branch vision transformer with the cross-attention mechanism and a graph pooling algorithm, CsAGP, for multimodal AD classification. We designed a multimodal feature fusion strategy based on the cross-attention mechanism to effectively learn the shared feature representation of MRI and PET images. Furthermore, a concise framework based on a graph pooling algorithm is developed to reduce token redundancy in the proposed model. Extensive experiments on the ADNI database demonstrate that the classification accuracy of our proposed CsAGP for AD vs. CN, AD vs. MCI, CN vs. MCI, and AD vs. CN vs. MCI are 99.04%, 97.43%, 98.57%, and 98.72%, which is 4.93%, 2.99%, 8.22% and 18.72% higher than current multimodal AD diagnosis methods, respectively.

The proposed CsAGP is slice-based and considers only axial view slices. Since 2D images cannot include all the information from a full brain scan. In addition, this study has not yet conducted a time processing comparison. Expanding the CsAGP for a full brain analysis and conducting comparative study on time processing will be a part of our future research.

## Figures and Tables

**Table 1:** The clinical information of the subjects.

Diagnosis	Number	Age	Gender(F/M)	MMSE	CDR
AD	214	75.1 ± 7.8	95/119	21.2 ± 4.1	0.9 ± 0.4
MCI	226	76.0 ± 7.4	82/144	25.6 ± 4.3	0.5 ± 0.3
CN	326	76.1 ± 6.4	165/161	28.7 ± 1.4	0 ± 0

**Table 2:** Classification results of the unimodal and multimodal method.

Auxiliary diagnosis	Modality	SEN (%)	SPE (%)	ACC (%)	AUC (%)
AD vs CN	MRIPETMRI + PET	96.7391.72**97.96**	98.3997.87**99.54**	97.8795.92**99.04**	99.6298.97**99.80**
AD vs MCI	MRIPETMRI + PET	92.2589.03**94.2**5	96.7296.33**98.81**	95.3794.12**97.43**	98.7998.27**99.23**
CN vs MCI	MRIPETMRI + PET	92.6494.61**98.52**	97.1094.77**98.61**	94.9494.69**98.57**	98.9298.83**99.76**
AD vs CN vs MCI	MRIPETMRI + PET	92.9692.28**98.65**	96.8896.49**99.34**	94.2193.37**98.72**	98.8298.24**99.86**

SEN: sensitivity; SPE: specificity; ACC: accuracy.

**Table 3:** Performance comparison of the different existing methods.

Tasks	Methods	SEN (%)	SPE (%)	ACC (%)	AUC (%)
AD vs CN	Fang et al (2020) (2020)	95.89	98.72	**99.27**	n/a
AD vs CN	Zhang et al (2019) (2019)	96.58	95.36	98.47	98.61
AD vs CN	Shi et al (2022) (2022)	96.10	97.47	96.76	97.03
AD vs CN	Song et al (2021) (2021)	93.33	94.27	94.11	n/a
AD vs CN	CsAGP (ours)	**97.96**	**99.54**	99.04	**99.80**
AD vs MCI	Fang et al (2020) (2020)	89.71	93.59	92.57	n/a
AD vs MCI	Zhang et al (2019) (2019)	90.11	91.82	85.74	88.15
AD vs MCI	Song et al (2022) (Song et al., 2021)	71.19	85.94	80.80	n/a
AD vs MCI	Liu et al (2022) (2022)	**94.91**	98.52	94.44	97.00
AD vs MCI	CsAGP (ours)	94.25	**98.81**	**97.43**	**99.23**
CN vs MCI	Fang et al (2020) (2020)	88.36	92.56	90.35	n/a
CN vs MCI	Zhang et al (2019) (2019)	97.43	84.31	88.20	88.01
CN vs MCI	Shi et al (2022) (2022)	85.98	70.90	80.73	78.75
CN vs MCI	Song et al (2022) (Song et al., 2021)	84.69	85.60	85.00	n/a
CN vs MCI	CsAGP (ours)	**98.52**	**98.61**	**98.57**	**99.76**
AD vs CN vs MCI	Song et al (2021) (2021)	55.67	83.40	71.52	n/a
AD vs CN vs MCI	Han et al (2020) (Kun et al., 2020)	n/a	n/a	67.74	n/a
AD vs CN vs MCI	Zhang et al (2022) (2022)	n/a	n/a	80.00	95.00
AD vs CN vs MCI	CsAGP (ours)	**98.65**	**99.34**	**98.72**	**99.86**

Bold value means the best indicator value under the same conditions and ‘n/a’ means no data.

**Table 4:** The classification results for different..r

r	SEN (%)	SPE (%)	ACC (%)	AUC (%)
0.1	95.61	98.04	96.30	99.32
0.3	95.56	98.42	97.00	99.49
0.5	98.65	99.34	98.72	99.83
0.7	98.69	99.36	98.79	99.86
0.9	**99.00**	**99.57**	**99.21**	**99.90**

**Table 5:** Ablations on FBLM and SFLM.

Method	FBLM*	SFLM*	FBLM	SFLM	SEN (%)	SPE (%)	ACC (%)	AUC (%)
A	✓	✓			98.49	99.25	98.23	99.86
B	✓			✓	98.46	99.20	98.47	99.79
C		✓	✓		98.51	99.27	98.63	99.81
D			✓	✓	**98.65**	**99.34**	**98.72**	**99.86**

**Table 6:** Classification results of removing CAFM.

Auxiliary diagnosis	SEN (%)	SPE (%)	ACC (%)	AUC (%)
AD vs CNw/o CAFM	**97.96**96.13	**99.54**98.44	**99.04**97.71	**99.80**98.71
AD vs MCIw/o CAFM	**94.25**93.00	**98.81**96.93	**97.43**96.05	**99.23**98.05
CN vs MCIw/o CAFM	**98.52**94.70	**98.61**96.60	**98.57**95.67	**99.76**99.12
AD vs CN vs MCIw/o CAFM	**98.65**95.30	**99.34**97.73	**98.72**95.70	**99.86**99.26

## Data Availability

The code will be available on https://github.com/weimingyang4/CsAGP after the article is accepted, and the authors do not have permission to share data. CsAGP: Detecting Alzheimer’s Disease from Multimodal Images via Dual-Transformer with Cross-Attention and Graph Pooling Anonymized.

## Footnotes

2 Available at https://adni.loni.usc.edu.

3 Available at https://fsl.fmrib.ox.ac.uk/fsl/fslwiki.

4 Available at https://github.com/ANTsX/ANTs.

5 Available at https://www.nitrc.org/projects/art.

6 Available at https://github.com/FNNDSC/med2image.
